# Shark Conservation: An Educational Approach Based on Children’s Knowledge and Perceptions toward Sharks

**DOI:** 10.1371/journal.pone.0163406

**Published:** 2016-09-29

**Authors:** Kwok Ho Tsoi, Sau Ying Chan, Yeung Chung Lee, Brian Ho Yeung Ip, Chi Chiu Cheang

**Affiliations:** Department of Science and Environmental Studies, The Education University of Hong Kong, Taipo, N.T., Hong Kong SAR; University of California Davis, UNITED STATES

## Abstract

Shark conservation has become a focus of current international conservation efforts. However, the misunderstanding of sharks and their negative public portrayal may hinder their conservation. More importantly, the consumption of shark fin, which is very common in Chinese cultures, poses a significant threat to sharks. Hong Kong has long been the world’s largest shark fin trading center. Shark conservation would become more sustainable if public understanding of this predatory fish and an appreciation of its ecological significance could be promoted. It is possible that the demand for fins could be effectively managed through long-term educational efforts targeted at younger generations. To provide essential baseline data for planning of these educational efforts, this project investigated the perceptions of 11 to 12 year-old primary school students in Hong Kong about sharks, and their understanding of ecological concepts and shark-related knowledge. The findings indicate that these students lack sufficient knowledge and possess misconceptions about sharks and their ecological significance in the marine ecosystem. The students’ conceptual understanding level is strongly correlated with their perceptions. Correlational analyses further demonstrated a positive association between formal education and perceptions toward shark conservation. The students who favoured shark fin consumption did so because of its tastiness, whereas concerns about shark population decline and the cruelty of shark hunting were the main reasons for not favoring shark fin consumption. This pilot study provides preliminary but important insights into primary school education regarding the conservation of sharks.

## Introduction

Studies have estimated that about 100 million sharks are killed every year [1]. The over-exploitation of sharks for the billion-dollar fin trade annually is driven by the high market demand for shark fins particularly in Asian regions, for ‘shark fin soup’ [2]. *Yu chi* (shark fin) is a traditional Chinese symbol of wealth and vitality and has become an important component of Chinese cuisine [3]. Shark fin soup is found on many Chinese restaurant menus in Hong Kong (S1 Fig). Due to this symbolic meaning and the rapid economic growth of mainland China, the expanding purchasing power of the Chinese has fueled the trade of this precious marine resource [4]. Hong Kong has long been recognized as the world’s largest shark fin importer [2], contributing at least half of the global trade in fins [1]. Clarke et al. [5] noted that Hong Kong is the major warehouse for shark fins in the mainland Chinese market. However, there are signs that the trading center for shark fins has shifted from Hong Kong to mainland China, resulting in uncertainty around monitoring and evaluation of the negative effects of shark consumption [2].

A recent study reported an 80% drop in sales by fin vendors in Guangzhou, China, and 85% of about 1600 Chinese respondents had given up fin consumption in the previous two years [6], implying success in the conservation of sharks to a certain degree. However, only 62% of the respondents attributed their decision to a desire “to protect sharks,” and slightly fewer avoided sharks due to concerns about the possibility of buying fake fins, contamination of the fins with mercury, and the government’s banning of shark fins from banquets [6]. Because the reduction in consumption is not solely driven by a perceived need to save sharks, it is possible that fin consumption may rebound if one day it is proved to be “safe”. As a result of prolonged harvesting, many shark species have experienced a rapid drop in their global populations over the past few decades [7], and a quarter of the world’s ray and shark species are considered threatened [8]. The conservation of these ecologically important species has become an international concern [8], as their decline exerts significant top-down effects on community structure and species diversity in marine habitats [9].

Shark conservation needs support from the general public [10], but reports abound that the public generally exhibits fear and negative attitudes toward large carnivorous animals [11], which may hinder conservation efforts. More importantly, the ecological role of sharks in the marine ecosystem is usually ignored [12] or misunderstood [13]. Alarming portrayals of sharks, particularly the great white, as some of the most feared and mysterious creatures on the planet, are widespread [14, 15]. In one study, children between 4 and 13 years of age perceived “jumping into a shark-infested pool” as one of the most fearful situations when they were asked to draw a risky situation in a draw-and-write investigation about how risk was perceived by these children [16]. Some children also personified sharks as “greedy, cunning and deceitful thieves and the swindler of the sea” [17], despite the fact that only six fatalities out of 98 unprovoked shark attacks were reported worldwide in 2015 [18].

The impediments to public engagement due to gaps between knowledge and action, and insufficient understanding of conservation practices [10] could be countered by knowledge and value development through education, but marine ecological issues are not specified in the science-related school curriculum [19]. Marine contexts are generally a minor area of discussion in comparison with terrestrial issues [20]. In a review of environmental themes in the General Studies curriculum for Hong Kong primary schools, only a few specifically marine contexts are elaborated under topics such as “The natural environment” or “Living things and their living environment” [21]. In a unit on “Love of nature” under the above topics, “Nature” is normally considered in the terrestrial rather than the marine environment. We hypothesize that the common misunderstandings and knowledge gaps associated with marine life conservation may be correlated to the lack of emphasis in the formal curriculum on marine life and marine environment.

From a constructivist perspective, children’s conceptions and perceptions on an issue will affect their interpretation of the world [22]. Ecological knowledge is regarded as the core foundation for promoting environmental education in children [23], and if the fundamental framework is not properly constructed, the learning of advanced concepts, particularly those regarding complicated ecological issues, will be hindered [24, 25]. Children who have misconceptions that originate from myths about an unfamiliar animal or incorrect information from the mass media are more likely to possess negative perceptions [13, 26]. In contrast, a better understanding of environmental issues is associated with more positive attitudes in children [27].

The aim of this study was thus three-fold: i) to investigate Hong Kong primary school students’ understanding of ecological concepts and their shark-related knowledge level and to explore their misconceptions about sharks; ii) to collect students’ perceptions or beliefs about this top predator; and iii) to examine any correlations among students’ conceptual knowledge, perceptions and beliefs, academic performance, and affection for sharks and preference for shark fin consumption. The questions and hypotheses of the study are presented below.

### Research questions

What is the level of ecological knowledge of senior primary school students about sharks, and what is their perception toward shark conservation? Are there any associations between the two?

### Hypotheses

1. Mastering ecological knowledge of sharks is associated with the development of a positive perception toward their conservation.
2. Formal education of the students supports
  - the development of a positive perception toward shark conservation;
  - the construction of ecological knowledge and concepts about sharks.
3. Personal affection for sharks is related to the students’ background knowledge and their perception of shark conservation.
4. The preference for eating shark fins is related to personal affection for sharks, perceptions toward shark conservation, and knowledge about the ecology of fish.

The results of this study will inform curriculum development with respect to conservation education for primary school students. We believe that using educational efforts to manage the demand for shark fins and the driving forces of the demand is a sustainable way to conserve sharks and marine environment.

## Materials and Methods

### Samples and pilot study

The study was approved by the Human Research Ethics Committee of the Hong Kong Institute of Education (now renamed The Education University of Hong Kong). Written informed consent was obtained from the school principals, teachers, students and their parents before the study. All information acquired from the study was kept anonymous to protect the participants’ privacy. A total of 137 Primary-6 (P6) students between 10 and 12 years of age (mean, 11 years) from two randomly selected primary schools (76 from School A; 61 from School B) in Hong Kong participated in this study. Two P6 classes from each school were randomly selected for participation.

The students’ ecological understanding and perceptions of shark conservation were assessed with a questionnaire. A pilot study was performed with eight students of similar age who were randomly selected from School C. The questionnaire was developed based on an analysis of their interviews. The external validity, precision, and reliability of the questionnaire were also assessed in the pilot study. The instrument was reviewed by four experts in science education, marine biology, and environmental science to ensure content validity. Minor revisions were made on the basis of the reviewers’ comments to ensure that the questions were comprehensible to primary students. Strong agreement among the reviewers on the relevance of these items to the instrument was reached (content validity index = 1; Kappa coefficient = 0.74), and the measurement tool showed a high level of reliability (reliability test: α = 0.854, n = 20).

### The questionnaire

A series of question items were developed to assess students’ perceptions and conceptual understanding of sharks. The questionnaire included 22 five-point Likert-type items (Tables 1 and 2). Both positive and negative wordings were used in the conceptual questionnaire items to minimize any acquiescent bias. For descriptive statistical analyses on positive statements, “strongly agree” scored 5 points and “strongly disagree” scored 1 point. The scores of the negatively worded items were reversed for inferential statistical analyses to accurately reflect the nature of the association between different variables. The option “neither agree nor disagree” was included to minimize the occurrence of random responses. The responses to both statement types were adjusted before any further data analysis. After the score adjustment, higher conceptual and perceptual scores represented a more precise understanding of ecological issues of sharks (with higher confidence) or a more positive perception toward shark conservation, respectively. In addition to these Likert-type questions, a number of issue-based items with multiple and non-conflicting options were constructed to reveal the children’s multi-dimensional views. The students were allowed to choose more than one response for this type of question.

**Table 1:** Frequency of response for each item about shark-related knowledge and ecological concepts.

			% Frequency of responses in Likert scale ^c^					Adjusted mean score (SD) ^d^^,^ ^e^		
								All respondents	Gender	
Item	Type ^a^	Concepts and knowledge ^b^	SA	A	NE	D	SD	(N = 137)	Boys (N = 70)	Girls (N = 67)
C1	-	Humans are the main diet of sharks.	0	1.5	12.4	26.3	59.9	4.45 ^f^ (0.77)	4.57 (0.73)*	4.31 (0.78)*
C2	-	Fewer sharks in the sea would enhance the fish catch.	2.9	11.7	18.2	27.7	39.4	3.89 (1.14)	4.00 (1.08)	3.78 (1.20)
C3	-	The decline of the shark population would not affect the ecosystem.	5.1	10.9	9.5	38.0	36.5	3.90 (1.17)	3.91 (1.18)	3.88 (1.16)
C4	-	The great white shark does not eat plankton, and the decline of the plankton population would not affect the great white shark.	2.9	16.8	39.4	21.9	19.0	3.37 (1.06)	3.36 (1.05)	3.39 (1.09)
C5	-	Too many sharks currently living in the ocean is the main cause of fish catch decline.	4.4	16.1	17.5	31.4	30.7	3.68 (1.19)	3.83 (1.22)	3.52 (1.16)
C6	-	The decline of the shark population would enhance the biodiversity of the marine ecosystem.	5.1	12.4	17.5	32.1	32.8	3.75 (1.19)	3.84 (1.15)	3.66 (1.23)
C7	-	The feeding mode of sharks can be shifted from carnivorous to herbivorous.	24.8	38.7	12.4	13.1	10.9	3.35 (1.28)	3.59 (1.27)*	3.10 (1.25)*
C8	+	Sharks play the role of predator in the marine ecosystem.	24.8	38.7	12.4	13.1	10.9	3.53 (1.30)	3.61 (1.30)	3.45 (1.29)
C9	+	The balance of the marine ecosystem would be upset if all sharks were wiped out from the sea.	54	25.5	8.0	7.3	5.1	4.16 (1.17)	4.37 (1.13)*	3.94 (1.17)*
		Mean of frequency	3.4	11.6	19.1	29.6	36.4	3.79 (0.54)	3.90 (0.53)*	3.67 (0.54)*
								Number of students who answered “agree” (%)		
C10		Diet type	Fish and other marine animals					135 (98.5)		
			Seaweed					22 (16.1)		
			Humans					33 (24.1)		
C11		Parties responsible for shark population decline	Shark catchers					98 (71.5)		
			Restaurant owners					58 (42.3)		
			Policy makers					55 (40.1)		
			Sharks					5 (3.6)		
			Fin consumers					83 (60.6)		

^*a*^ ‘+’ denotes positive statement, ‘−’ denotes negative statement.

^*b*^ Items C1 through C9 are issues with 5-point conflicting options, and items C10 and C11 are issues with multiple non-conflicting options.

^*c*^ SA, strongly agree; A, agree to some extent; NE, neutral; D, disagree to some extent; SD, strongly disagree.

^*d*^ Mean and SD of scores for each conceptual-based issue.

^*e*^ Asterisk (*) denotes significant difference (*p* < 0.05).

^*f*^ Scores are adjusted. Higher conceptual scores represent more precise understanding of ecological issues of sharks.

**Table 2:** Frequency of response for each issue and the perception toward shark conservation.

			% Frequency of responses in Likert scale ^b^					Adjusted mean score (SD) ^c^^,^ ^d^		
								All respondents	Gender	
Item	Type ^a^	Perception of sharks	SA	A	NE	D	SD	(N = 137)	Boys (N = 70)	Girls (N = 67)
P1	−	Sharks like killing marine organisms.	10.2	24.1	5.1	43.8	16.8	3.33^e^ (1.29)	3.51 (1.24)*	3.13 (1.33)*
P2	−	The appearance of sharks is associated with coming misfortune.	2.2	3.6	13.1	35	46	4.19 (0.95)	4.27 (0.87)	4.10 (1.03)
P3	−	Fish would live happily if sharks disappeared.	7.3	12.4	5.8	39.4	35	3.82 (1.24)	3.94 (1.19)	3.70 (1.29)
P4	−	Human beings are frequently attacked by sharks.	5.8	9.5	17.5	31.4	35.8	3.82 (1.19)	3.81 (1.12)	3.82 (1.27)
P5	−	Killing sharks is good for the safety of swimmers.	0.7	2.2	3.6	22.6	70.8	4.61 (0.73)	4.70 (0.55)	4.51 (0.88)
P6	−	Killing sharks is a punishment for bullying marine organisms.	0.7	1.5	2.9	23.4	71.5	4.64 (0.69)	4.69 (0.60)	4.58 (0.76)
P7	−	Sharks are tyrants in the ocean.	5.8	11.7	9.5	37.2	35.8	3.85 (1.20)	3.99 (1.22)	3.72 (1.17)
P8	−	The ocean world would be peaceful without sharks.	1.5	5.1	5.1	35	53.3	4.34 (0.90)	4.39 (0.87)	4.28 (0.93)
P9	−	Swimming in the sea would be safe if the sea had no more sharks.	5.8	27.7	7.3	30.7	28.5	3.48 (1.32)	3.60 (1.30)	3.36 (1.33)
P10	−	“Sharks eat humans, humans eat sharks” is fair.	0.7	6.6	13.1	19.7	59.9	4.31 (0.98)	4.24 (0.92)	4.39 (1.04)
P11	−	The ocean would be full of life without sharks.	3.6	4.4	8	32.1	51.8	4.24 (1.03)	4.27 (1.03)	4.21 (1.02)
		Mean of frequency	4	9.9	8.3	31.9	45.9	4.06 (0.67)	4.13 (0.65)	3.98 (0.68)

^*a*^ ‘+’ denotes positive statement, ‘−’ denotes negative statement.

^*b*^ SA, strongly agree; A, agree to some extent; NE, neutral; D, disagree to some extent; SD, strongly disagree.

^*c*^ Mean and SD values of scores for each perception-based issue.

^*d*^ Asterisk (*) denotes significant difference (*p* < 0.05).

^*e*^ Scores are adjusted. Higher perceptual scores represent more positive perceptions toward shark conservation.

To reveal the affection of students for sharks and their preference for fin soup consumption, dichotomous-choice items with possible rationales presented as multiple and non-conflicting options were provided in the questionnaire. A lesson period of 35 minutes was provided for the students to complete the questionnaire. After completing the questionnaire, the students were asked to draw a picture of a shark to provide a visual representation of their perception of sharks.

### Scoring and data analysis

The conceptual understanding and perceptions of the students were quantified by generating mean conceptual and perceptual scores for each Likert-type item in the questionnaire (Tables 1 and 2). The overall means for the conceptual and perceptual items were also computed. The students’ score for the General Studies (GS) examination in the first semester was adopted as an indicator of academic performance. GS is the principal subject in which Hong Kong primary students acquire knowledge about wildlife conservation through the formal curriculum. The scores were standardized into *z*-scores with respect to school-based means. The *z*-scores of the two schools were pooled for subsequent analyses. Responses to the dichotomous-choice items on affection for sharks and preferences for shark fin consumption were analyzed using contingency table analysis to determine factor association. Descriptive statistical data and relationships among variables were analyzed using PASW Statistics 18.0.0 software (SPSS, Inc.). Pearson’s correlation analysis was performed to determine the strength and direction of the correlations between pairs of variables. The Spearman test was adopted instead in case of assumption violation. Regression analysis was performed to determine causal relationships among different variables.

To further reveal the relationship between the conceptual and perceptual scores, the respondents were categorized into high-score (score ≥ mean) and low-score (score < mean) groups. Independent *t*-tests were used to test for significant differences in the perceptual scores between the two conceptual score groups. A similar method was applied to the analysis of GS subject scores.

## Results

### Ecological concepts and shark-related knowledge

The mean value of the conceptual scores on the 5-point Likert scale was 3.79 (Table 1). A score between 3 and 4 means that the students exhibit a generally, but not completely, correct idea. They still have some reservations or uncertainties that require further resolution. A high score (above 4) indicates the students’ high confidence in recognizing the concept. This information has educational significance in terms of which concepts should be given more attention and which require a strengthening strategy. A 5-point Likert scale can provide more information than a dichotomous scale (true or false) for this purpose. Reliability tests revealed an acceptable Cronbach’s alpha score (α = 0.60), indicating a satisfactory level of internal consistency and reasonable intercorrelation. The gender differences in the mean conceptual score are statistically significant (*t* (135) = 2.50, *p* = 0.014).

The students showed a good understanding that humans are not the main diet of sharks with the highest conceptual score in C1, 4.45 (*mean score*). In contrast, the students performed the worst on the issue of a shifting feeding mode (C7, 3.35, Table 1), revealing a misconception that sharks can be herbivorous. Similarly, 16% of the students believed that sharks’ diet is seaweed, and more than 30% did not realize the predatory role of the sharks (C8). Only 20% of the students disagreed with the statement “the great white shark does not eat plankton, and the decline of the plankton population would not affect the great white shark,” indicating their poor understanding of biological interactions within the ecological system. The students’ moderate understanding is also revealed by the finding that more than 60% believed the decline in both fish catch (C5, 3.68) and biodiversity in the marine ecosystem (C6, 3.75) are caused by “too many sharks.” Most of the students considered shark catchers were the most responsible for the decline of shark (72%) while fin consumers were the second accountable party (61%). Despite these findings, about 80% agreed that the balance of the ecosystem would be interrupted if sharks were removed from the ecosystem (C9, 4.16).

### Perceptions toward shark conservation

The mean value of perceptual scores was 4.06 (Table 2). The internal consistency of the responses was high (α = 0.85). No significant difference was found between the two genders (*t* (131) = 1.53, *p* = 0.129). The general perception toward shark conservation was positive (score > 4.00). The students scored highest on P5 (4.61) and P6 (4.64), indicating that they generally disagreed with killing sharks to ensure safe swimming or punishing sharks for “bullying” marine organisms (Table 2). Nevertheless, it is worth noting that 34% of the children had the impression that sharks “like” killing marine organisms (Table 2). This perception seems to be consistent with their drawings (S2 Fig), which portray sharks invariably as a fish with a big mouth opened wide and armed with rows of sharp and triangular teeth ‘fiercely’. As shown by their responses to the item “Swimming in the sea would be safe if the sea had no more sharks” (P9, 3.48), the children still considered sharks as the main threat to swimmers in the sea.

### Comparison between conceptual and perceptual scores

**Fig 1:**
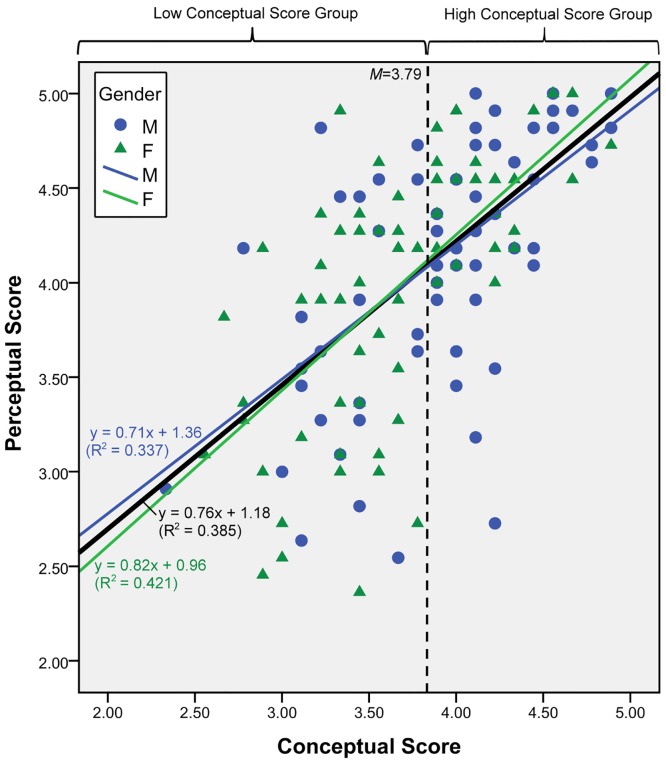
Relationship between ecological concepts and shark-related knowledge (knowledge score) and perceptual view of primary school students (perceptual score). Regression equation of best-fit line for all respondents is *y* = 0.76*x* + 1.18 (*R*^2^ = 0.385, *p* < 0.001). High and low conceptual score groups were differentiated at mean score of 3.79.

A strongly positive correlation between the students’ ecological and shark-related knowledge and their perceptions of sharks was found for both boys (*r* = 0.602, *n* = 70, *p* < 0.01) and girls (*r* = 0.672, *n* = 67, *p* < 0.01). Because the slopes of the best-fit lines for boys and girls did not differ significantly based on regression analysis (*t* = 0.661, *p* = 0.510), the data for the two genders were combined. The regression equation of the best-fit line for all respondents was determined to be *y* = 0.76*x* + 1.18 (Fig 1; *R*^2^ = 0.385, *p* < 0.001). The analysis also showed a strong positive correlation between the conceptual and perceptual scores (*r* = 0.634, *p* < 0.01) for the combined data. The difference between the mean perceptual score of the high conceptual score group (*m* (SD) = 4.42 (0.46), n = 68) and that of the low score group (*m* (SD) = 3.70 (0.65), n = 69) was statistically significant (*t* (123) = 7.59, *p* < 0.0005) (Fig 1). This finding supports our first hypothesis that students’ ecological knowledge of sharks is associated with the development of a positive perception toward sharks.

### Correlation between conceptual/perceptual scores and academic performance

**Fig 2:**
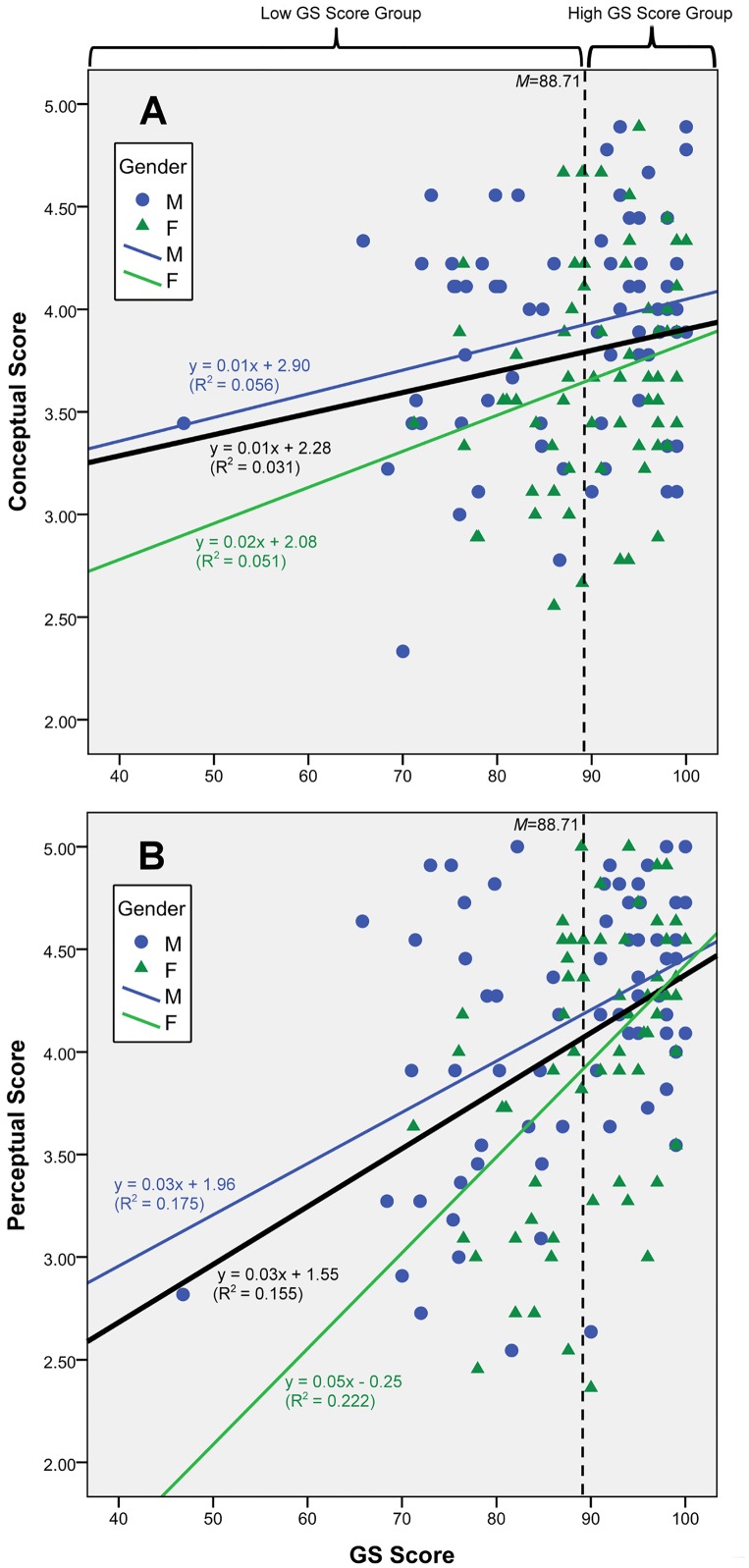
Relationship between final subject scores for General Studies and conceptual or perceptual scores. High and low conceptual score groups were differentiated at mean score of 88.71. (A) Conceptual score. Regression equation of best-fit line for all respondents for conceptual score is *y* = 0.01*x* + 2.28 (*R*^2^ = 0.031, *p* < 0.05). (B) Perceptual score. Regression equation of best-fit line is *y* = 0.03*x* + 1.55 (*R*^2^ = 0.155, *p* < 0.001).

The mean GS subject scores, which were taken as an indicator of the students’ academic performance, were compared with their perceptual scores (Fig 2). The mean conceptual scores of the high subject score group (*m* (SD) = 3.88 (0.53), n = 81) and the low subject score group (*m* (SD) = 3.65 (0.54), n = 56) were statistically significant (*t* (135) = 2.54, *p* < 0.05). The difference in the mean perceptual score between the high subject score group (*m* (SD) = 4.27 (0.54), n = 81) and the low subject score group (*m* (SD) = 3.74 (0.72), n = 56) was also statistically significant (*t* (96) = 4.69, *p* < 0.0005). Correlation analyses further demonstrated a weak positive correlation between the students’ conceptual scores and academic performance (Pearson, *r* = 0.176, n = 137, *p* < 0.05; Spearman, *r* = 0.163, *p* = 0.057) and a medium to strong positive correlation between the students’ perceptual scores and academic performance (Pearson, *r* = 0.393, n = 137, *p* < 0.01).

The regression equation of the best-fit lines for all participants between their subject *z*-score and conceptual score is *y* = 0.034*x* + 3.79 (*R*^2^ = 0.004, *p* = 0.475), and that between their *z*-score and perceptual score is *y* = 0.131*x* + 4.057 (*R*^2^ = 0.038, *p* < 0.05) (data not shown). The analysis shows no significant correlation between the students’ conceptual scores and *z*-score (Pearson, *r* = 0.062, *p* = 0.475; Spearman, *r* = 0.041, *p* = 0.634); however, a statistically significant positive correlation was obtained between the students’ perceptual scores and subject *z*-scores (Pearson, *r* = 0.195, *p* < 0.05). Tests on both the raw GS scores and the standardized *z*-scores obtained concordant results; academic performance exhibited a positive correlation with the students’ perceptual view, but not their conceptual understanding about sharks and related ecological issues. The students’ academic performance, which is an indication of the influence of formal education, has a significant association with the development of a positive perception toward shark conservation but not with ecological knowledge, indicating that hypotheses 2(i) is supported, whereas hypothesis 2(ii) is rejected.

### Relationship between conceptual/perceptual scores and affection for sharks and preference for shark fin consumption

**Fig 3:**
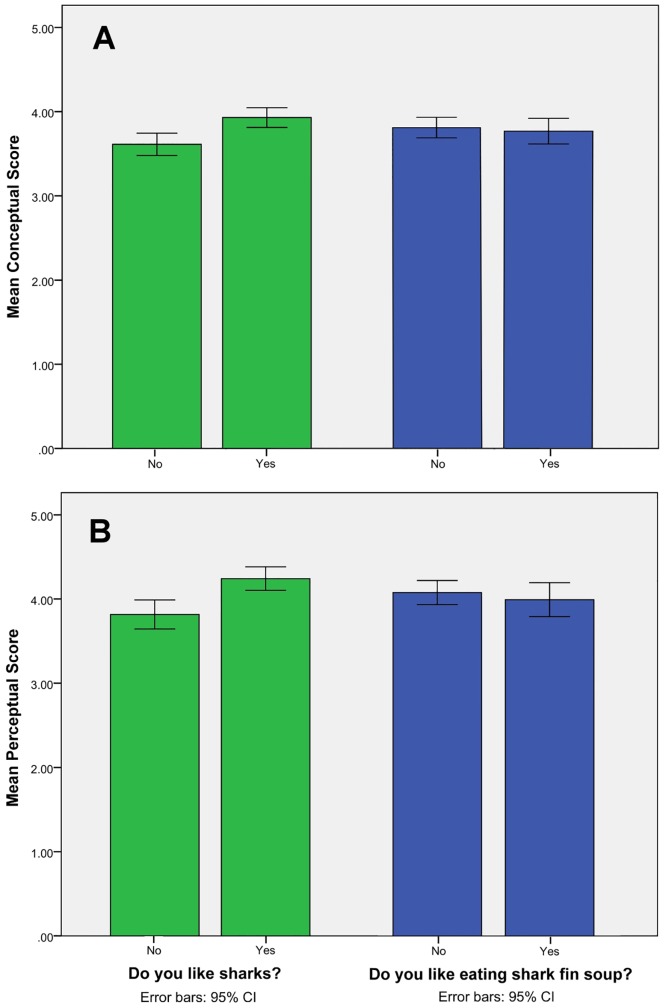
Mean scores of students who said they “like sharks” or “don’t like sharks,” and “like eating shark fin soup” or “don’t like eating shark fin soup.” (A) Conceptual score. (B) Perceptual score (*p < 0.05).

About 56% of the students indicated “I like sharks” (Table 3, n = 135). The difference in the conceptual scores of the “like” (*m* (SD) = 3.93 (0.51)) and “don’t like” (*m* (SD) = 3.61 (0.51)) groups is statistically significant (*t* (133) = 3579, *p* < 0.001) (Fig 3A), with the “like” group showing a higher conceptual score. The difference in the mean perceptual scores of the “like” group (*m* (SD) = 4.24 (0.61)) and the “don’t like” group (*m* (SD) = 3.82 (0.67)) is also statistically significant (*t* (133) = 3.85, *p* < 0.001) (Fig 3B), with the “like” group again having a higher perceptual score. Thus, the third hypothesis that personal affection for sharks is associated with both the students’ knowledge and their perception toward shark conservation is supported.

**Table 3:** Students’ affection for sharks and preferences for shark fin soup consumption and their associated reasons.

		Frequency (%) [% of the students choosing the reasons among those who expressed “I like eating shark fin soup”]		Frequency (%) [% of the students choosing the reasons among those who expressed “I don’t like eating shark fin soup”]	N
Preferences	“I like sharks” ^a^	75 (55.6)	“I don’t like sharks” ^a^	60 (44.4)	135
	“I like eating shark fin soup” ^b^	46 (35.4)	“I don’t like eating shark fin soup” ^b^	84 (64.6)	130
Reasons	Tasty	40 (30.8) [87.0]	Not tasty	24 (18.5) [28.6]	130
	Nutrient rich	21 (16.2) [45.7]	Not nutritive	6 (4.6) [7.1]	130
	Good for the body	14 (10.8) [30.4]	Harmful to the body	1 (0.8) [1.2]	130
	Preciousness	12 (9.2) [26.1]	Too extravagant	16 (12.3) [19.0]	130
	Important food ingredient in traditional events	11 (8.5) [23.9]	Shark population decline	56 (43.1) [66.7]	130
			Cruelty	61 (46.9) [72.6]	130

Students were allowed to choose more than one reason for their preference.

^*a*^ Two students who did not indicate their preferences toward sharks were excluded.

^*b*^ Seven students who selected both preference options were excluded from statistics.

However, more than 35% indicated “I like eating shark fin soup” (Table 3, n = 130). No obvious association was observed between “like/dislike sharks” and “like/dislike eating shark fin soup” (χ^2^ test = 0.790, *p* > 0.05; phi value = 0.079, *p* > 0.05). The difference in the conceptual scores of the “like eating” group (*m* (SD) = 3.72 (0.51)) and the “don’t like eating” (*m* (SD) = 3.81 (0.56)) group is not statistically significant (*t* (128) = 0.09, *p* > 0.05) (Fig 3A), and neither is the difference in the perceptual scores of the “like eating” (*m* (SD) = 3.82 (0.67)) and “don’t like eating” groups (*m* (SD) = 4.08 (0.66)) (*t* (128) = 0.70, *p* > 0.05) (Fig 3B). Hence, the fourth hypothesis that a personal preference for eating fins is associated with students’ perceptions or ecological knowledge is not supported. Among those who “like eating” shark fin, the main reasons are its “tastiness” (87.0%), and it is “nutrient rich” (45.7%). For those who dislike eating shark fin soup, “cruelty” (72.6%) and “shark population decline” (62.7%) were their main concerns (Table 3).

## Discussion

### Ecological knowledge of sharks demonstrated by primary school students in Hong Kong

Ecological knowledge is the foundation of children’s environmental education [23, 28]. Strong knowledge of ecological concepts and positive attitudes toward the animals of concern are at the root of conservation success. Our results affirm that the strength of students’ ecological knowledge of sharks is associated with their perception toward shark conservation, albeit with the uncertain causal relationship. On one hand, the students’ learning of shark-related knowledge could enhance their awareness on shark conservation and nurture their pro-shark attitude. On the other hand, the students with pro-shark attitude, which may be previously influenced by their family or other contextual factors, could be more eager to learn about shark or other pro-environmental information. In fact, some relevant studies have demonstrated the influence of factual knowledge about the organisms to be conserved on the attitude of the students towards the organisms. Makashvili et al. [29] have pointed out the role of knowledge in reducing the negative image of the snake among the junior undergraduates in Georgia. Soga et al. [30] have found that vicarious experience of the nature could also boost up the willingness of children to conserve biodiversity. While the causal relationship between conceptual and perceptual views on shark conservation could not be addressed by the present study, further intervention studies could shed lights on the causality between conception and perception of the students.

Nevertheless, this positive relationship between knowledge and attitude among students was also observed by other researchers. Prokop and Tunnicliffe [26] found that more alternative conceptions (misconception) observed towards the bats would lead to a more negative attitude. Torkar et al. [31] revealed that a better factual knowledge basis was associated with the more positive attitudes of the secondary students in Slovenia towards Eurasian Otter. Our study shows that Hong Kong children have insufficient knowledge and have misconceptions about sharks and their ecological role, echoing reports (e.g., [26, 32]) that misconceptions and problematic conceptual frameworks on environmental issues exist among young children. Additionally, feeding or trophic issues are in general poorly understood [13, 33, 34] as evidenced by the confusion over the ecological role of sharks as top predators and their feeding modes.

Feeding relationships are generally regarded as critical determinants of understanding ecosystems [33, 34]. The respondents realized the mutual influence of two organisms in a direct predator-prey relationship but neglected the interdependency of other organisms in the same habitat (e.g., [33, 35]). The students’ misconception that sharks compete with humans for fish yield further supports this. A previous study found that more than 30% of 9-year-old students failed to realize the complicated interactions in the ecosystem [13]; this limitation of understanding appears to have persisted as children grow older. This learning challenge is persistent across the different education levels and is widespread. Barman and Mayer [36] explored children’s similar misconception that the removal of producers from the ecosystem would not affect shark populations. This implies that elucidating the feeding relationships in a complicated ecosystem is an important first step in promoting children’s ecological knowledge which is believed to be correlated to the development of positive attitudes to shark conservation.

Other than the ecological system, students believed “shark catchers” rather than “fin eaters” should take greater responsibility for the decline in shark populations. This may suggest that the children failed to consider more complex second order relationships in this multiple-intercalating issue. This simplistic reasoning of causal relationships is similar to the thinking the students exhibited in considering the predator-prey relationships in a food chain, as discussed in the previous paragraph. This reasoning is not appropriate for understanding complicated ecological systems in which many biotic components interact [37]. This simplistic reasoning may also create difficulties in learning complex issues involving multiple causes [37, 38].

Groves and Pugh [39] indicated that children always think in a simple and direct way. Many studies have also revealed that students are used to considering environmental issues that have been highly simplified [33, 35, 38, 40, 41], leading to difficulty in grasping the concepts of mutual causality to explain complicated feeding relationships [37, 38]. This echoes the finding that children apparently fail to consider environmental issues from a holistic perspective [37, 40, 42]. As suggested by Jordan et al. [23], a deeper understanding of the interactions and connections could be promoted if the concepts of “systems and cycles” were enhanced. Eilam [43] has emphasized the importance of understanding the interaction among the components within the ecosystem and establishing a network for integrating all these components into a systematic model. In light of these findings, the schools should be assisted to construct a curriculum framework in primary ecological studies, with incorporation of basic ecological knowledge as the foundation on which to build a more complicated food-web model that emphasizes feeding relationships in addition to the simplified food chain approach.

The students’ shaky understanding of ecology is further underscored by their contradictory beliefs that marine ecosystems would be upset if all sharks were wiped out (C9) while at the same time believing that a decline in shark populations would enhance the biodiversity of the marine ecosystem (C6). This reflects the difficulty children have in understanding the complex roles of sharks in the marine ecosystem. In terms of Bloom’s taxonomy of cognitive outcomes [44], the students in this study have only achieved superficial comprehension; they fell short of applying ecological knowledge to make sense of the dynamic changes in individual populations in the ecosystem.

### Perceptions and affection for sharks and preference for fin soup consumption

The average perceptual score (> 4.00) in our study does not support the view that the public has a negative view of sharks (e.g., [11, 16, 17]). Most of the senior primary school students did not agree with killing sharks. However, it is worth noting that some students still felt the threat of the big fish in the sea as evidenced by their perception that “human beings are frequently attacked by sharks” (P4) (30%), with a similar proportion believing that “swimming in the sea would be safe if the sea had no more sharks” (P9) and that “sharks ‘like’ killing marine organisms.” This affective response may reflect what the students understand about sharks. The students’ drawings of sharks (S2 Fig) also seem to reflect their impression of sharks as the fearsome predators portrayed by the mass media.

For the minority of students who like to eat shark fins, “tastiness” appeared to be the major driver of their choice. Additionally, the belief that shark fins are “precious” and an “important food ingredient in traditional events” seems to be firmly embedded in some of these students’ minds. This echoes a recent study that revealed that about 8% of 1000 public interviewees did not accept the exclusion of shark fin soup from Chinese wedding banquets, which are the most frequent occasions for consuming the soup [45]. This has important implications for the future market demand for sharks when these children become potential shark fin consumers, particularly because we found that they believe “shark catchers” rather than “fin eaters” are more responsible for the exploitation of sharks.

The majority of the students in this study did not like eating shark fin, and most of them were aware of the decline of shark populations and the cruelty of shark hunting, which seems encouraging for conservation. This echoes a study on the social attitudes toward shark fin consumption in Cantonese diners, which found that about 66% of more than 1000 interviewees felt uncomfortable consuming such an endangered animal [46]. However, a caveat regarding these positive findings is that only a very small proportion of the children realized that shark fin is not nutrient rich and could be harmful to humans due to heavy metal contamination [47]. This indicates that the children were not aware of issues surrounding this ‘food’ that have been quite well reported, implying a lack of recognition of these issues by schools and parents.

### Educational implications for shark conservation

Our findings indicate that children’s negative attitudes toward sharks may be associated with their lack of understanding of the role of sharks in the ecosystem, as documented in other studies [11, 26, 48]. The positive correlation between students’ academic performance and their perceptual score, however, may imply that the General Studies curriculum is more effective in fostering “respect and care for all living things” and recognizing “the importance of environmental conservation” [21] than it is at promoting students’ understanding of ecological systems. These positive learning outcomes seem to have been achieved with the specific support of curriculum instruction [21] and teachers’ efforts.

Further study is needed to decipher any causal relationship between the curriculum contents and teaching approaches, and the development of students’ conceptual understanding and positive attitudes toward biological conservation. As argued by Jordan et al. [23], a deeper understanding of the interactions and connections of organisms within an ecosystem could be promoted if relevant concepts were explicitly presented in form of “systems” and “cycles.” We therefore recommend the construction of a more effective conceptual foundation for environmental education in primary schools by incorporating a simple food-web model into the currently adopted food chain model, for teaching of feeding relationships and ecology-related topics. This would enhance students’ deeper reasoning on the indirect or second-order relationships that threaten particular animal species, and ultimately biodiversity. In addition, the development of students’ critical thinking or analytical skills and promotion of their understanding and application of knowledge could enhance students’ understanding of ecological system and counter the influences of emotive reactions triggered by sensational media reports or myths. Increasing the public’s knowledge about sharks can help gain support for shark conservation [49]. Many studies have revealed that people who consider themselves to be knowledgeable about shark conservation are more likely to adopt pro-shark behavior [49, 50]. Gallagher et al. [50] explained the level of subjective knowledge about shark conservation is a significant variable on identifying the threatened species in fishing practice. The knowledgeable anglers will express more understanding about the shark conservation and will be willing to select a fishing method on reducing the impact of damaging sharks. However O’Bryhim and Parsons [49] observed the level of knowledge about sharks being acquired are significantly varied among different sources. While the aforementioned examples illustrated that the acquisition of knowledge, even though in the form of subjective knowledge, would greatly affect the pro-shark behavior, we argue that the strengthening of the school curriculum would support the improvement of positive attitude, or even behavior, in children towards shark conservation on saying ‘no’ to the shark fin soup in their future.

In the long run, the issue of shark hunting could be resolved by reducing market demand, which requires conscious and persistent educational efforts. Students should be explicitly informed that shark fin consumption is the significant driving force for the indiscriminate killing of sharks and adverse interference with the marine ecosystem. Such educational strategies could be applied to promote the conservation of other vulnerable animals that are illegally hunted due to great market demand for ornamental or medicinal use. Despite its limited sample size, this study can be considered an important pilot study that paves the way for more detailed and focused studies in this area. Future studies could be extended to compare the views of students from different cities and regions, including Macau, mainland China, Taiwan, and Southeast Asia, in which shark fins are in high demand, for exploring the potential causes in the various associations of the educational dimensions. More importantly, the elucidation of the way on how to improve the educational approach for promoting the perception towards shark conservation is the next focus, such as the strengthening of the conservation and ecological education in school curriculum.

## Supporting Information

S1 Fig A wedding banquet menu extracted from the webpage of a Chinese restaurant in 2016 (the name of the restaurant is not shown). (TIF)

S2 Fig Representative drawings of sharks by the primary school students. (TIF)
