# Supplementary figures and images for: Shark Conservation: An Educational Approach Based on Children’s Knowledge and Perceptions toward Sharks
Source: PLoS One. 2016 Sep 29;11(9):e0163406. doi: 10.1371/journal.pone.0163406 (PMC5042495; doi:10.1371/journal.pone.0163406)

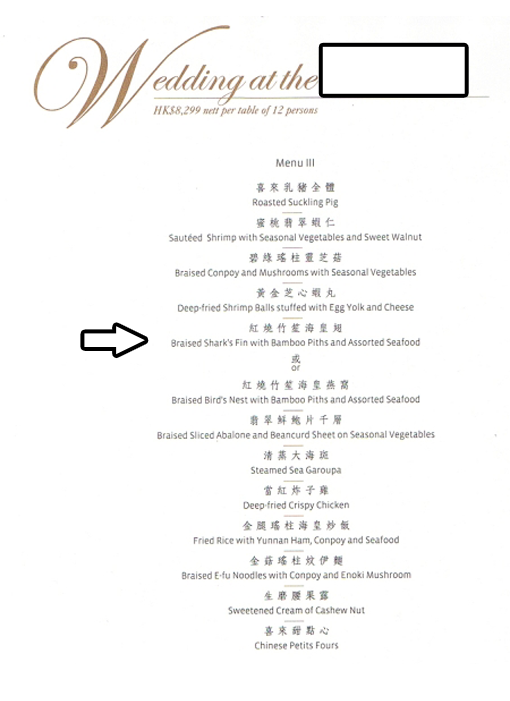

Supplement: S1 Fig — (TIF) [file pone.0163406.s001.tif]

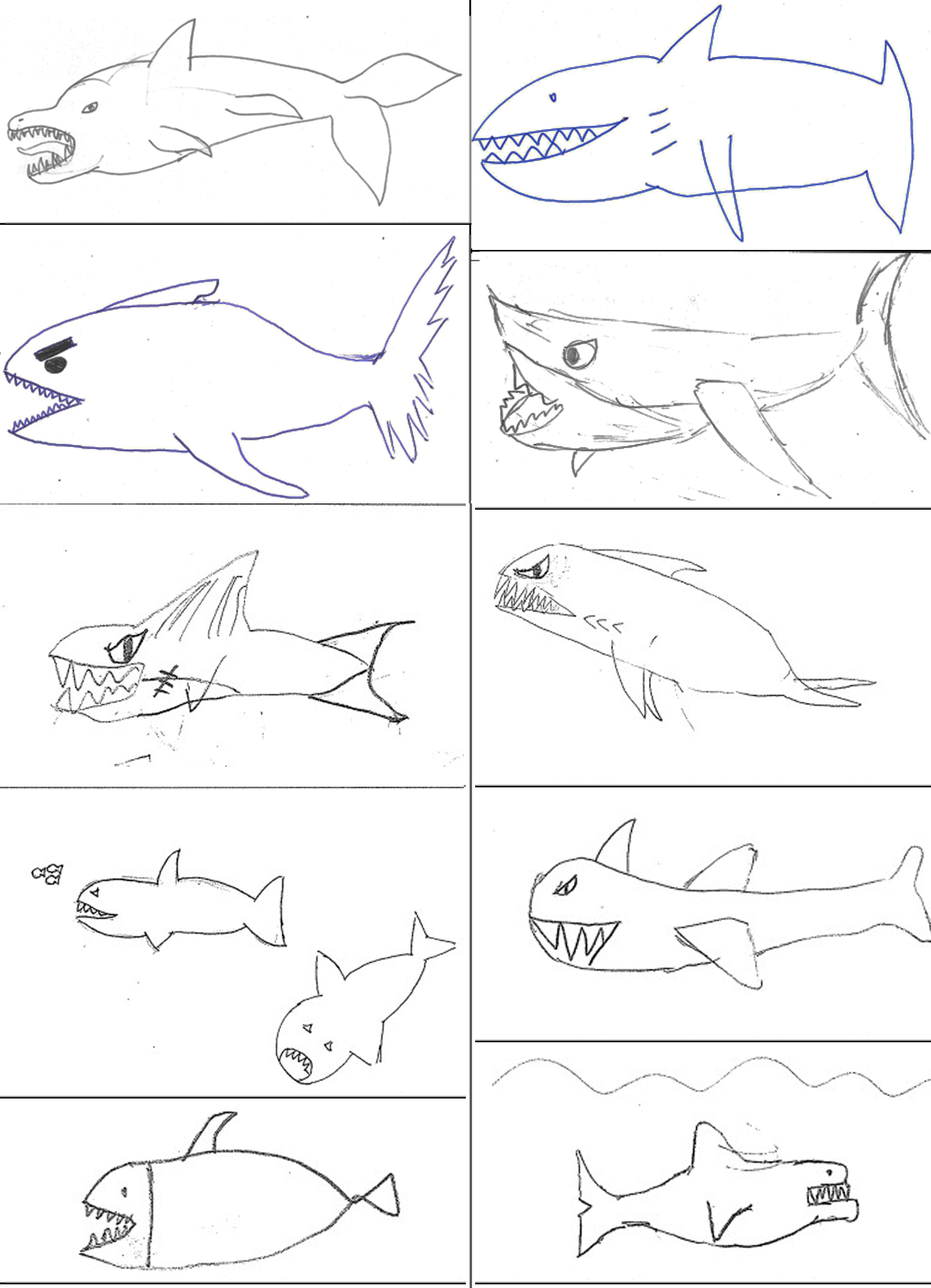

Supplement: S2 Fig — (TIF) [file pone.0163406.s002.tif]
